# A class II 5-enolpyruvylshikimate-3-phosphate synthase from *Pseudomonas* P818 confers robust glyphosate tolerance in transgenic plants

**DOI:** 10.1016/j.abiote.2026.100023

**Published:** 2026-01-17

**Authors:** Yuhao Su, Jie Cao, Yan Liu, Xiaodong Xie, Tianyong Zhao, Gaoyi Cao, Yunjun Liu

**Affiliations:** a State Key Laboratory of Crop Gene Resources and Breeding, Institute of Crop Sciences, Chinese Academy of Agricultural Sciences, Beijing, 100081, China; b National Key Laboratory of Crop Improvement for Stress Tolerance and Production, College of Life Sciences, Northwest A&F University, Yangling, 712100, China; c Tianjin Key Laboratory of Intelligent Breeding of Major Crops, College of Agronomy & Resources and Environment, Tianjin Agricultural University, Tianjin, 300392, China; d Hotan Vocational and Technical College, Hetian, 848000, China

**Keywords:** *Pseudomonas* P818, 5-enolpyruvylshikimate-3-phosphate synthase, Glyphosate tolerance, Transgenic plants

## Abstract

Glyphosate is a widely used herbicide that targets 5-enolpyruvylshikimate-3-phosphate synthase (EPSPS), blocking the shikimate pathway and leading to plant death. The discovery of novel EPSPS genes is key to engineering glyphosate tolerance in crops. In this study, we isolated the glyphosate-tolerant bacterial strain *Pseudomonas* P818 from glyphosate-contaminated soil and cloned its class II EPSPS gene (*818-EPSPS*). Sequence and phylogenetic analyses revealed typical motifs of class II EPSPS. Kinetic characterization revealed a high *K*i/*K*m ratio (10.4), indicating that 818-EPSPS has low affinity for glyphosate (high *K*i value) while retaining high catalytic efficiency (low *K*m value). The heterologous expression of *818-EPSPS* restored growth in *E. coli* strain ER2799 under glyphosate stress, confirming its functional resistance. Transgenic *Arabidopsis thaliana* and tobacco (*Nicotiana tabacum*) plants heterologously expressing *818-EPSPS* exhibited strong tolerance to glyphosate, maintaining growth at concentrations that were lethal to the wild-type controls. We introduced codon-optimized *818-EPSPS* into maize (*Zea mays*), generating stable transgenic plants. Transgenic maize line EP03, carrying a single-copy insertion, showed robust tolerance to up to four times the recommended dosage of glyphosate in field trials. Genomic sequencing revealed that the T-DNA in EP03 was inserted into chromosome 5 without disrupting any host genes. Our findings establish *818-EPSPS* as a promising candidate for engineering glyphosate-tolerant crops and provide a new genetic resource for maize improvement and biosafety applications.

*Dear Editor*,

Glyphosate (N-phosphonomethyl glycine) is one of the most widely used herbicides in agriculture due to its broad-spectrum efficacy against weeds, low toxicity in mammals, and relatively limited environmental persistence. Glyphosate inhibits 5-enolpyruvylshikimate-3-phosphate synthase (EPSPS), which catalyzes the conversion of phosphoenolpyruvate (PEP) and shikimate-3-phosphate (S3P) to 5-enolpyruvylshikimate-3-phosphate (EPSP) in the sixth step of the shikimate pathway, leading to depletion of aromatic amino acids and ultimately plant death [1]. Following the identification of EPSPS as the primary target of glyphosate in the 1980s, EPSPS quickly became the focus of efforts to develop transgenic glyphosate-tolerant crops.

Despite extensive efforts, only two EPSPS-derived technologies have achieved widespread commercial success in transgenic glyphosate-resistant crops: *CP4* EPSPS and the *E. coli* EPSPS TIPS mutant [2,3]. Nevertheless, soil microorganisms, particularly those in extreme environments, harbor a wealth of genetic resources that remain underexplored and may provide novel genes for agricultural applications [2,[4], [5], [6]]. Several bacterial *EPSPS* genes (*G2 EPSPS*, *HTG7 EPSPS*, *A1501 EPSPS*, and *RD EPSPS*) were cloned from *Pseudomonas fluorescens* [7], *Halomonas variabilis* [8], *Pseudomonas stutzeri* [9], and uncultured soil bacteria [10], respectively. *AM79 EPSPS*, which was cloned from uncultured soil bacteria, represents a good candidate for the development of transgenic glyphosate-tolerant crops [11]. In China, two glyphosate-tolerant lines have obtained biosafety certificates: transgenic soybean (*Glycine max*) line Zhonghuang6106, which overexpresses *G2-EPSPS* and *GAT* genes; and transgenic cotton (*Gossypium hirsutum*) line GGK2, which overexpresses *AM79-EPSPS* and *GAT* genes (https://www.moa.gov.cn/ztzl/zjyqwgz/). The discovery of new EPSPS genes is crucial for facilitating the development of new glyphosate-tolerant crop varieties.

In this study, we isolated glyphosate-tolerant bacterial strains from glyphosate-contaminated soil samples collected near a glyphosate-manufacturing facility in Zhejiang Province, China. Twenty-eight colonies grew well on plates containing 300 mM glyphosate (Fig. 1A). Amplification and sequencing of *16S rRNA* revealed that these strains belonged to *Enterobacteriaceae*, *Pseudomonas*, *Arthrobacter*, and *Acinetobacter*, among others. We selected *Pseudomonas* strain P818 for further characterization. *A1501-EPSPS* [9] and *AM79-EPSPS* [11] are known to enhance glyphosate tolerance. Therefore, to evaluate the glyphosate tolerance of strain P818, we selected recombinant ER2799 strains carrying *A1501-EPSPS* and *AM79-EPSPS* as controls. ER2799 is an *aroA*-deficient mutant strain whose function can be restored by introducing *A1501-EPSPS* or *AM79-EPSPS*. However, under high glyphosate stress, both control strains exhibited impaired growth. By contrast, strain P818 grew robustly under the same conditions (Fig. 1B). These findings indicate that strain P818 could serve as a valuable resource for investigating the mechanisms of glyphosate tolerance and for discovering new glyphosate-tolerance genes.

**Fig. 1:**
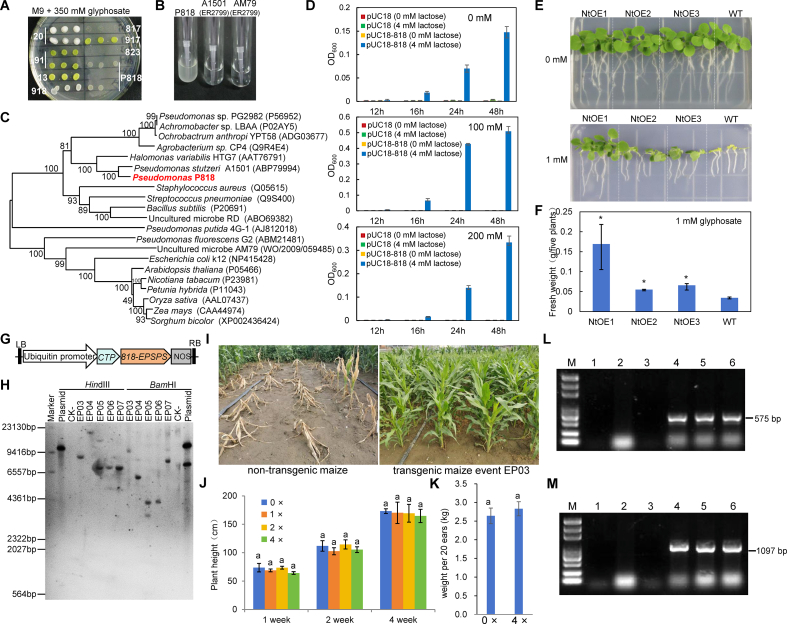
Cloning of *818-EPSPS* and analysis of its ability to confer glyphosate tolerance in transgenic plants. **A** Growth of different bacterial strains on M9 medium supplemented with 350 mM glyphosate; **B** Growth of *Pseudomonas* strain P818 and ER2799 recombinants expressing the glyphosate-resistance genes *A1501* and *AM79* in liquid M9 medium containing 300 mM glyphosate; **C** Phylogenetic analysis of EPSPS proteins from strain P818 and other species; **D** Growth curves of 818-EPSPS recombinant strains on different concentrations of glyphosate; **E** Phenotypes of transgenic tobacco heterologously expressing *818-EPSPS* grown for 10 days on MS medium without (top) and with 1 mM glyphosate (bottom); **F** Fresh weight of transgenic tobacco plants grown for 10 days on MS medium supplemented with 1 mM glyphosate; asterisks indicate significant differences between the transgenic lines and WT; **G** T-DNA region of p3301UbiSp818, which was used for maize transformation; **H** Southern blot analysis of transgenic maize events; **I** Representative phenotypes of transgenic EP03 plants one week after spraying with glyphosate; **J** Plant height of transgenic maize line EP03 at 1, 2, and 4 weeks after spraying with 0-, 1-, 2-, and 4-times the recommended field dosage of glyphosate. The same letter above the columns indicates no significant difference; **K** Ear weight of transgenic maize plants sprayed with 0- and 4-times the recommended field dosage of glyphosate; **L** Event-specific PCR of 5′-flanking sequences of transgenic maize event EP03; **M** Event-specific PCR of 3′-flanking sequences of transgenic maize event EP03. M, molecular weight markers.

To amplify the *EPSPS* gene in *Pseudomonas* P818, we aligned conserved domains of various EPSPS-encoding genes and designed an anchor primer and degenerate primers for thermal asymmetric interlaced PCR (TAIL-PCR) [12]. Following two rounds of TAIL-PCR, distinct bands of approximately 1.0 kb were obtained (Fig. S1). Sequence analysis showed that *818-EPSPS* contains a 1323 bp open reading frame with a G+C content of 65 %, encoding a 440 amino-acid protein. Phylogenetic analysis indicated that 818-EPSPS is a class II EPSPS, which contains five domains typical of this class (Fig. 1C, Fig. S2). At the amino-acid level, 818-EPSPS shared the highest identity with *Pseudomonas stutzeri* A1501 (86.82 %) [9] but lower similarity with CP4 EPSPS (50.91 %) [2] (Fig. S2; Table S1).

EPSPS variants with low affinity for glyphosate (high *K*i value) that maintain high catalytic efficiency (low *K*m value) can confer high glyphosate resistance to crops. CP4 EPSPS, which has been used in glyphosate-tolerant maize and soybean, has a high *K*i/*K*m value of 32 [13]. AM79-EPSPS (also known as GR79-EPSPS), which has been used in transgenic cotton [14] and transgenic maize [13], has a *K*i/*K*m value of 10.6 [13]. To evaluate the potential use of 818-EPSPS for developing glyphosate-tolerant crops, we characterized its kinetic parameters. 818-EPSPS exhibited a *K*m of 8.31 μM and a *K*i of 86.28 μM, resulting in a *K*i/*K*m ratio of 10.4, which is similar to that of AM79 EPSPS but lower than that of CP4 EPSPS [2]. Given that AM79 EPSPS was successfully used to develop transgenic cotton line GGK2, the high *K*i/*K*m value of 818-EPSPS suggests it is well-suited for conferring glyphosate tolerance in crops.

To assess the ability of *818-EPSPS* to confer glyphosate tolerance, we cloned this gene into the *Bam*HI/*Sal*I sites of pUC18 to generate pUC18-818, which we introduced into the *aroA*-deficient *E. coli* strain ER2799. ER2799 carrying the empty pUC18 vector served as the control. Control strains harboring pUC18 failed to grow in M9 medium with or without lactose. Strains expressing *818-EPSPS* also failed to grow in M9 medium without lactose; however, they exhibited robust growth in M9 medium with lactose (Fig. 1D). Even under increasing glyphosate concentrations, the *818-EPSPS* transformants maintained substantial growth, although growth was partially inhibited at higher concentrations (Fig. 1D). These findings demonstrate that *818-EPSPS* confers glyphosate tolerance in bacteria.

To further evaluate the potential of *818-EPSPS* for developing glyphosate-tolerant transgenic crops, we generated transgenic tobacco (*Nicotiana tabacum*) and Arabidopsis (*Arabidopsis thaliana*) plants heterologously expressing this gene and examined their glyphosate tolerance. We fused the signal peptide sequence from the pea ribulose-1,5-bisphosphate carboxylase (*rbcS*) small subunit to *818-EPSPS* to target 818-EPSPS to the chloroplast. Both the signal peptide and the *EPSPS* gene were controlled by the CaMV 35S promoter and cloned into pCAMBIA3301 for plant transformation (Fig. S3). For *Arabidopsis*, we germinated seeds from three independent T_3_ homozygous lines (AtOE1, AtOE2, AtOE3) and wild-type (WT) plants on MS medium supplemented with or without 0.5 mM glyphosate and examined the phenotypes of the resulting seedlings. No phenotypic differences were observed in the absence of glyphosate. However, in the presence of 0.5 mM glyphosate, the transgenic seedlings remained green and continued to grow, whereas WT seedlings displayed severe chlorosis and inhibited growth (Fig. S4). For tobacco, the growth of transgenic and WT plants was comparable on control medium. By contrast, 1 mM glyphosate caused severe chlorosis and death of WT seedlings, whereas transgenic plants expressing *818-EPSPS* retained green leaves and sustained growth (Fig. 1E). Biomass measurements confirmed that the transgenic lines exhibited significantly higher fresh weight than WT plants under glyphosate stress (Fig. 1F). Collectively, these results indicate that heterologous overexpression of *818-EPSPS* confers strong glyphosate tolerance in both *Arabidopsis* and tobacco, highlighting its potential utility in engineering glyphosate-tolerant crops.

To generate maize with enhanced glyphosate tolerance, we constructed a codon-optimized version of *818-EPSPS* for maize (designated as *m818-EPSPS*). We constructed p3301UbiSp818, a plant expression vector producing the *Arabidopsis* chloroplast transit peptide 2 fused to the N-terminus of m818-EPSPS, with transcription driven by the maize *Ubiquitin* promoter (Fig. 1G). We transformed immature maize embryos with *Agrobacterium tumefaciens* strain LBA4404 carrying p3301UbiSp818 and grew them on selection medium with high concentrations of glyphosate to generate transgenic seedlings with high glyphosate tolerance. We obtained three hundred and fourteen independent transformants. PCR analysis using m818-EPSPS-specific primers confirmed the successful integration of the gene (Fig. S5).

The copy number and transcript levels of *EPSPS* genes can affect glyphosate resistance in some plants [[15], [16], [17], [18]]. The successful generation of transgenic crops often relies on introducing one or a few copies of *EPSPS* driven by a strong constitutive promoter to achieve stable resistance while minimizing the risk of gene silencing, metabolic burden, or unpredictable insertional effects. To further characterize the *EPSPS* integration patterns in the transgenic maize lines, we performed Southern blot analysis of five representative lines (EP03, EP04, EP05, EP06, and EP07). A single band was detected in EP03, EP04, EP06, and EP07 following *Hin*dIII and *Bam*HI digestion, indicating the presence of single-copy insertions, whereas EP05 displayed two bands, suggesting a two-copy insertion (Fig. 1H). We evaluated the glyphosate tolerance of these maize lines in the field. We sprayed transgenic plants at the 5–7 leaf stage with glyphosate at 0, 1-, 2-, and 4-times the recommended commercial dosage. Approximately one week after treatment, all WT plants were killed, whereas the transgenic plants exhibited normal growth (Fig. 1I). Measurement of plant height at 1, 2, and 4 weeks post-treatment revealed no significant differences among transgenic plants treated with different concentrations of glyphosate (Fig. 1J; Fig. S6). No significant difference in ear weight was observed between the glyphosate-treated and control groups (Fig. 1K). These results demonstrate that these transgenic maize lines exhibit strong tolerance to high doses of glyphosate and represents a promising candidate for the development of glyphosate-tolerant maize cultivars.

Given the potential breeding value of these lines, we chose EP03 for further analysis. We examined the sequences flanking the T-DNA insertion site using third-generation sequencing. The exogenous T-DNA was inserted in reverse orientation into the maize genome at chr5:187,073,989, with 333 bp of the maize genomic sequence integrated at the 3′ end. To specifically detect and authenticate the transgenic event EP03, we designed two pairs of specific primers to amplify the extension of the LB- and RB-flanking sequences, each including part of the maize genome and part of the T-DNA. We detected the expected specific PCR products in EP03 plants but not in non-transgenic plants (Fig. 1L and M). These results suggest that both pairs of specific primers could be used to specifically detect and authenticate the transgenic event EP03 and its derivatives.

Taken together, our findings establish 818-EPSPS as a functionally efficient enzyme that confers glyphosate tolerance. Transgenic maize harboring single-copy insertions exhibited strong and stable herbicide tolerance, underscoring the utility of *818-EPSPS* in crop biotechnology. Future work should explore stacking *818-EPSPS* with other herbicide-tolerance gene or genes involved in metabolic detoxification pathways to further enhance the durability of glyphosate resistance in field settings.

## 1 Materials and methods

### 1.1 Isolation and identification of glyphosate-resistant bacterial strain P818

Soil samples contaminated with glyphosate were collected near a glyphosate storage site at Zhejiang Wynca Chemical Industrial Co., Ltd. (Zhejiang Province, China). Soil suspensions were prepared in 0.9 % (w/v) NaCl solution and plated on M9 solid medium containing 50 mM glyphosate at 10^−6^ dilution. Colonies resistant to glyphosate were screened after two days of incubation at 37 °C. Resistant clones were purified by repeated transfer to fresh M9 plates containing glyphosate.

### *1.2* Isolation of the *818-EPSPS* gene

Chromosomal DNA was extracted from *Pseudomonas* strain P818 using an EasyPure™ Genomic DNA Kit (Beijing TransGen Biotech). Thermal asymmetric interlaced PCR was performed to amplify the *EPSPS* gene [12]. An anchor primer (aroA-A,GTAATACGACTCACTATAGGCATGGCGATGCGATGATC) was designed based on conserved *EPSPS* sequences, while a degenerate primer (aroA-S,GTAATACGACTCACTATAGGAADMGNCCDWTDRR) incorporated variable bases. PCR was carried out using the following program: 93 °C for 2 min, 95 °C for 1 min, 10 cycles of 94 °C for 30 s, 68 °C for 30 s, 72 °C for 1 min, followed by 12 cycles of 4 combined cycles as follows: 94 °C for 30 s, 30 °C for 2 min, 72 °C for 1 min, 94 °C for 30 s, 68 °C for 30 s, 72 °C for 1 min, 94 °C for 30 s, 68 °C for 30 s, 72 °C for 1 min, 94 °C for 30 s, 50 °C for 30 s, 72 °C for 1 min; and a final extension at 72 °C for 10 min. The PCR product was purified and sequenced.

### 1.3 Sequence alignment and homology modeling

Multiple sequence alignment was performed using MEGA with default parameters [19]. The alignment output was exported as a formatted file and subsequently processed and adjusted using GeneDoc [20].

### 1.4 Determination of the Ki/Km value

EPSPS activity was assayed as previously described [11] with minor modifications. For Michaelis–Menten analysis, the S3P concentration was fixed at 1 mM, while the PEP concentration varied from 0, 0.5, 0.67, 1, 2, 5, to 10 mM (dilution ratios 0, 1:19, 1:14, 1:9, 1:4, 1:1, and undiluted, respectively). The enzyme was added last to start the reaction, and the time-staggered color development sequence described above was followed.

Initial velocities (v) were obtained based on phosphate formation under the linear time window. Hanes–Woolf plots were constructed according to:

$$\frac{\left[S\right]}{v}=\frac{1}{V_{\max}}\left[S\right]+\frac{K_{m}}{V_{\max}}$$

From linear regression, the slope equals 1/*V*_max_, the y-intercept equals *K*_*m*_/*V*_max_, and the x-intercept equals −*K*_*m*_. *V*_max_ was derived from the ratio of the y-intercept to the slope.

Glyphosate inhibition was assessed at inhibitor concentrations of 0, 0.5, 1, and 2 mM. Initial rates were measured at PEP concentrations of 66.7, 100, 200, and 500 μM under each inhibitor level. Apparent *K*_*m*_ and *V*_max_ values were obtained from Hanes–Woolf plots, and *K*_*i*_ (glyphosate) was estimated accordingly. Data are presented as mean ± SD unless otherwise indicated. Linear regressions for Hanes–Woolf plots were performed using standard least-squares fitting. Replicate outliers due to pipetting or timing errors (e.g., deviation from the 6-s staggering) were excluded based on pre-specified QC rules.

### 1.5 Vector construction

The *818-EPSPS* gene was amplified by PCR and cloned into the *Bam*HI/*Sal*I sites of pUC18 to construct pUC18-818.

To construct the vector for tobacco and *Arabidopsis* transformation, a stop codon–deleted *818-EPSPS* fragment was generated by PCR and fused with an HA tag sequence. This fragment was cloned into the *Bam*HI/*Sac*I sites of p3301-121spAM79 [11] to generate p3301-121sp-818HA.

To construct the vector for maize transformation, *818-EPSPS* was codon-optimized with maize-preferred codons and cloned into pCAMBIA3301 to construct p3301UbiSp818, in which the signal peptide sequence of *Arabidopsis* chloroplast targeting peptide 2 was fused in front of the codon-optimized *m818-EPSPS* gene, with transcription controlled by the maize *Ubiquitin* promoter.

### *1.6* Evaluation of glyphosate tolerance in *E. coli* strain ER2799

Glyphosate tolerance of *E. coli* strain ER2799 was evaluated as previously described [11] with some modifications. ER2799 competent cells were transformed with pUC18-aroA818 and plated on LB medium containing 50 μg/mL ampicillin. Positive clones were cultured, harvested, and resuspended in M9 medium to OD_600_ = 0.5. Aliquots (500 μL) were inoculated into 200 mL M9 liquid medium containing 0, 100, or 200 mM glyphosate with or without lactose (4 mM). Cell density (OD_600_) was measured after incubation for 12 h, 16 h, 24 h, and 48 h. Each experiment was performed in triplicate. ER2799 containing empty pUC18 served as the vector control.

### 1.7 Plant transformation

Tobacco transformation was performed as previously described [21] using the phosphinothricin (PPT) gene as the antibiotic marker gene. Transgenic plants were confirmed by PCR amplification of *818-EPSPS* and transferred to a greenhouse for harvesting and analysis. *Arabidopsis* transformation was performed using the traditional floral dip method [22]. For maize transformation, the vector was transferred into *Agrobacterium tumefaciens* strain LBA4404, which was introduced into immature embryos of maize inbred line Zong31 as described previously [23].

### 1.8 Analysis of the glyphosate tolerance of transgenic plants

Glyphosate resistance in T_1_ transgenic tobacco seedlings was evaluated as previously described [11]. Plant injury was recorded and fresh weight was measured. For *Arabidopsis*, surface-sterilized T_2_ seeds were germinated on MS medium containing 0.5 mM glyphosate and grown vertically for 10 days under a 16 h light/8 h dark cycle at 100 μmol m^−2^ s^−1^.

Transgenic maize plants were planted in the field. Plants at the 5–7 leaf stage were sprayed with 0, 0.9, 1.8, and 3.6 kg ha^−1^ glyphosate. Plant height was measured at 1, 2, and 4 weeks after glyphosate treatment to evaluate glyphosate tolerance. At physiological maturity, ears were harvested, and their weight was recorded as a measure of ear yield.

### 1.9 Southern blot analysis

For Southern blot analysis of transgenic maize plants containing *m818-EPSPS*, a 561 bp probe for *m818-EPSPS* was amplified using the primers 5′-GTCCAAGAGGCCGATGAACA-3′ and 5′-GGGATGTCGATGCCCTTCAG-3′. Approximately 100 μg genomic DNA from transgenic plants and the non-transgenic controls was digested with *Hin*dIII or *Bam*HI. The digested DNA was separated by electrophoresis on 0.8 % agarose gels and transferred to a nylon membrane (Amersham). The membrane was hybridized with DIG-labeled probes using a DIG High Primer DNA Labeling and Detection Starter Kit III (Roche). Hybridization was carried out according to the instruction manual.

## CRediT authorship contribution statement

**Yuhao Su:** Investigation. **Jie Cao:** Investigation. **Yan Liu:** Methodology. **Xiaodong Xie:** Validation, Methodology. **Tianyong Zhao:** Writing – review & editing, Conceptualization. **Gaoyi Cao:** Writing – review & editing, Writing – original draft, Investigation, Conceptualization. **Yunjun Liu:** Writing – review & editing, Writing – original draft, Conceptualization.

## Declaration of competing interest

The authors declare that they have no known competing financial interests or personal relationships that could have appeared to influence the work reported in this paper.

## Data Availability

The data are available from the corresponding author Yunjun Liu upon reasonable request.
