# Supplementary material for: A class II 5-enolpyruvylshikimate-3-phosphate synthase from Pseudomonas P818 confers robust glyphosate tolerance in transgenic plants
Source: aBIOTECH. 2026 Jan 17;7(2):100023. doi: 10.1016/j.abiote.2026.100023 (PMC12973396; doi:10.1016/j.abiote.2026.100023)
Supplement: Multimedia component 1 [file mmc1.docx]

**Supplemental Table S1. Analysis of Amino Acid Sequence Identity among EPSPS Proteins**

|  | P818 | E.coli k12 | AtEPSPS | NtEPSPS | ZmEPSPS | SbEPSPS | OsEPSPS | A1501 | G2 | HTG7 | 4G-1 | CP4 | RD | AM79 |
| --- | --- | --- | --- | --- | --- | --- | --- | --- | --- | --- | --- | --- | --- | --- |
| P818 | 100 | 34.54 | 36.97 | 33.41 | 33.33 | 34.35 | 33.88 | 86.82 | 39.35 | 70.91 | 32.69 | 50.91 | 50 | 37.26 |
| E coli k12 | 34.54 | 100 | 57.75 | 57.51 | 54.69 | 56.57 | 57.41 | 34.3 | 40.69 | 38.26 | 33.49 | 36.23 | 33.66 | 42.58 |
| AtEPSPS | 36.97 | 57.75 | 100 | 76.02 | 74.7 | 76.04 | 75.05 | 35.85 | 41.47 | 38.12 | 38.31 | 26.74 | 35.87 | 44.42 |
| NtEPSPS | 33.41 | 57.51 | 76.02 | 100 | 73.56 | 76.44 | 75.54 | 32.71 | 42.92 | 37.41 | 36.63 | 33.57 | 34.52 | 44.98 |
| ZmEPSPS | 33.33 | 54.69 | 74.7 | 73.56 | 100 | 92.81 | 86.48 | 33.41 | 41.38 | 35.05 | 35.99 | 31.55 | 32.86 | 43.51 |
| SbEPSPS | 34.35 | 56.57 | 76.04 | 76.44 | 92.81 | 100 | 87.72 | 33.65 | 41.61 | 36.07 | 37.53 | 31.71 | 33.57 | 43.74 |
| OsEPSPS | 33.88 | 57.41 | 75.05 | 75.54 | 86.48 | 87.72 | 100 | 33.1 | 40.46 | 35.6 | 36.71 | 32.02 | 33.33 | 45.08 |
| A1501 | 86.82 | 34.3 | 35.85 | 32.71 | 33.41 | 33.65 | 33.1 | 100 | 33.09 | 69.55 | 31.41 | 49.09 | 50.7 | 37.35 |
| G2 | 39.35 | 40.69 | 41.47 | 42.92 | 41.38 | 41.61 | 40.46 | 33.09 | 100 | 36.32 | 35 | 35.66 | 32.91 | 42.75 |
| HTG7 | 70.91 | 38.26 | 38.12 | 37.41 | 35.05 | 36.07 | 35.6 | 69.55 | 36.32 | 100 | 29.55 | 50.23 | 48.6 | 34.6 |
| 4G-1 | 32.69 | 33.49 | 38.31 | 36.63 | 35.99 | 37.53 | 36.71 | 31.41 | 35 | 29.55 | 100 | 30.79 | 32.76 | 33.82 |
| CP4 | 50.91 | 36.23 | 26.74 | 33.57 | 31.55 | 31.71 | 32.02 | 49.09 | 35.66 | 50.23 | 30.79 | 100 | 46.01 | 31.67 |
| RD | 50 | 33.66 | 35.87 | 34.52 | 32.86 | 33.57 | 33.33 | 50.7 | 32.91 | 48.6 | 32.76 | 46.01 | 100 | 32.92 |
| AM79 | 37.26 | 42.58 | 44.42 | 44.98 | 43.51 | 43.74 | 45.08 | 37.35 | 42.75 | 34.6 | 33.82 | 31.67 | 32.92 | 100 |

**
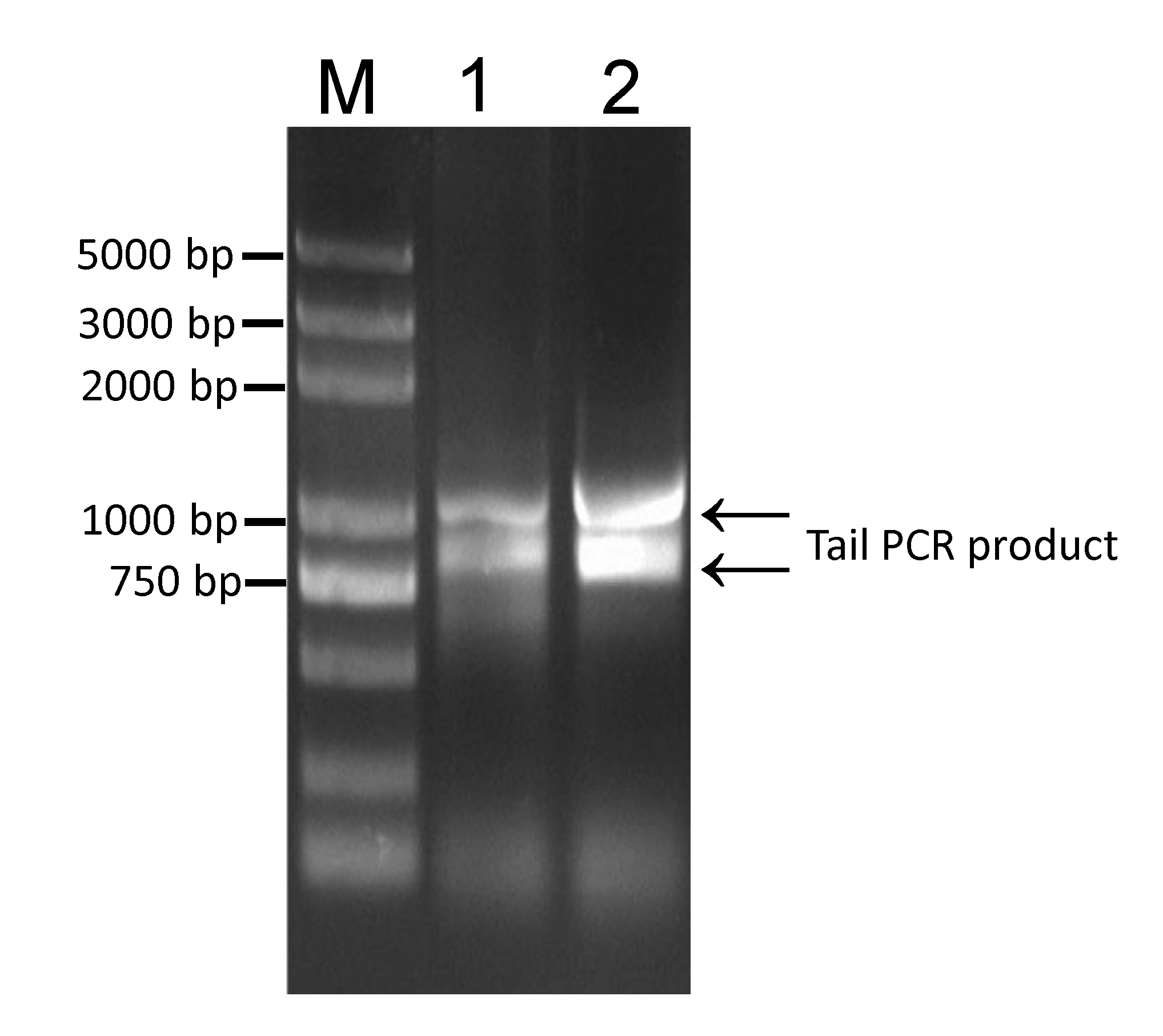
**

**Supplemental Figure S1. The EPSPS coding gene of strain P818 was amplified using the tail-PCR**

M：*Trans*2K^®^ Plus DNA Marker；1-2：Genomic DNA of isolate strain 818

**
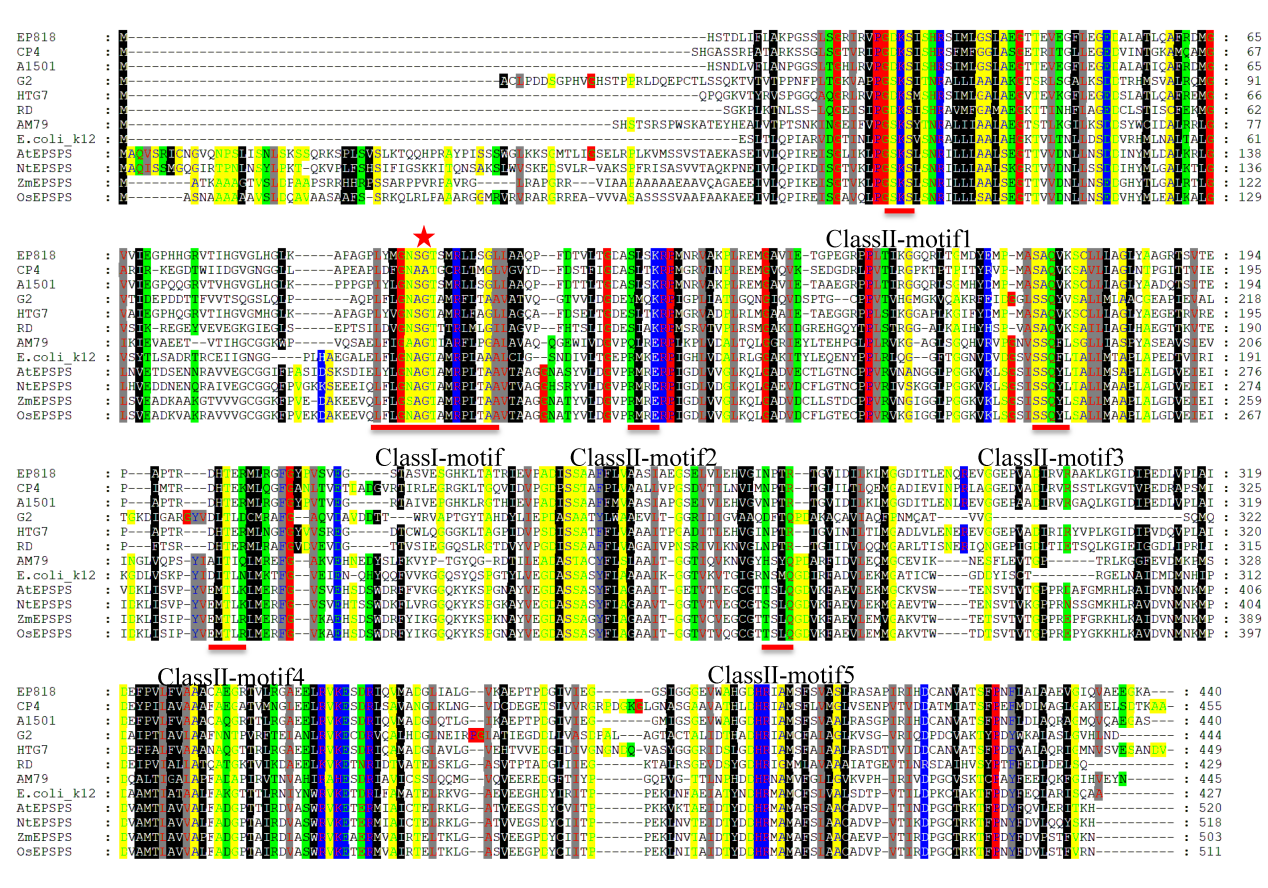
**

**Supplemental Figure S2. Multiple sequence alignment and conserved region analysis of EPSPS**

Multiple sequence alignment was conducted for 818-EPSPS alongside other Class I and Class II EPSPS proteins. The Class I motif corresponds to the conserved amino acid domain located at residues 95–106 in Class I EPSPS, while Class II motifs 1–5 represent the five characteristic conserved domains of Class II EPSPS. An asterisk indicates the amino-terminal alanine at position 100 (Ala100), numbered according to the CP4 EPSPS sequence.

**
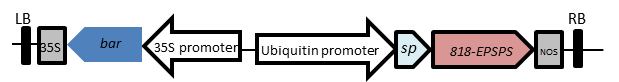
**

**Supplemental Figure S3. The T-DNA region of plasmid p3301-818-EPSPS**

LB, T-DNA left border; 35S, CaMV 35S terminator; 35S promoter, CaMV 35S promoter; Ubiquitin promoter, maize ubiquitin promoter; sp,signal peptide sequence from the pea ribulose-1,5-bisphosphate carboxylase (*rbcS*) small subunit; 818-EPSPS, 818-EPSPS gene; NOS, nos terminator; RB, T-DNA right border.


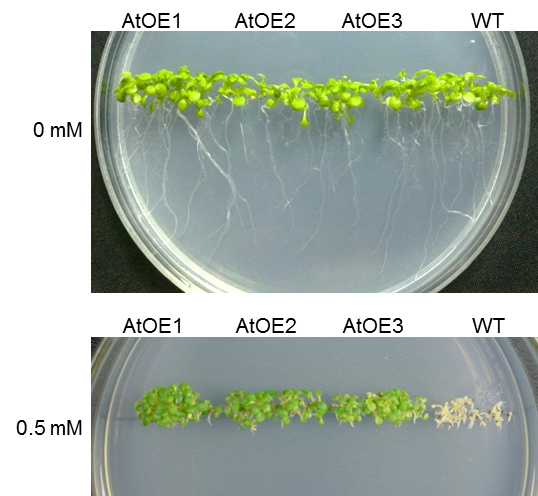


**Supplemental Figure S4. Phenotypes of Arabidopsis grown for 11 days on MS medium without (top) or with 0.5 mM glyphosate (bottom)**

**
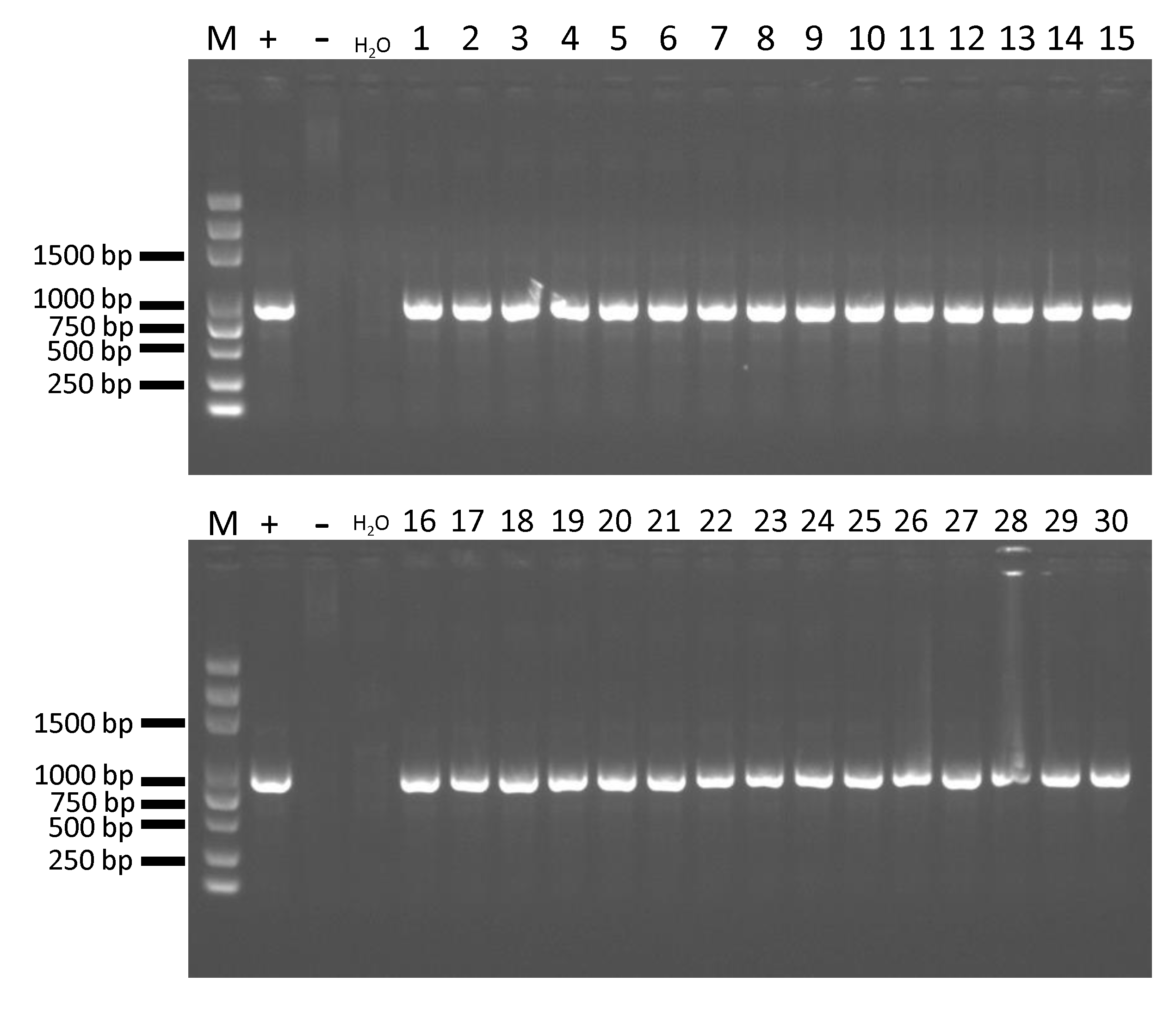
**

**Supplemental Figure S5. PCR analysis of transgenic maize plants**.

**
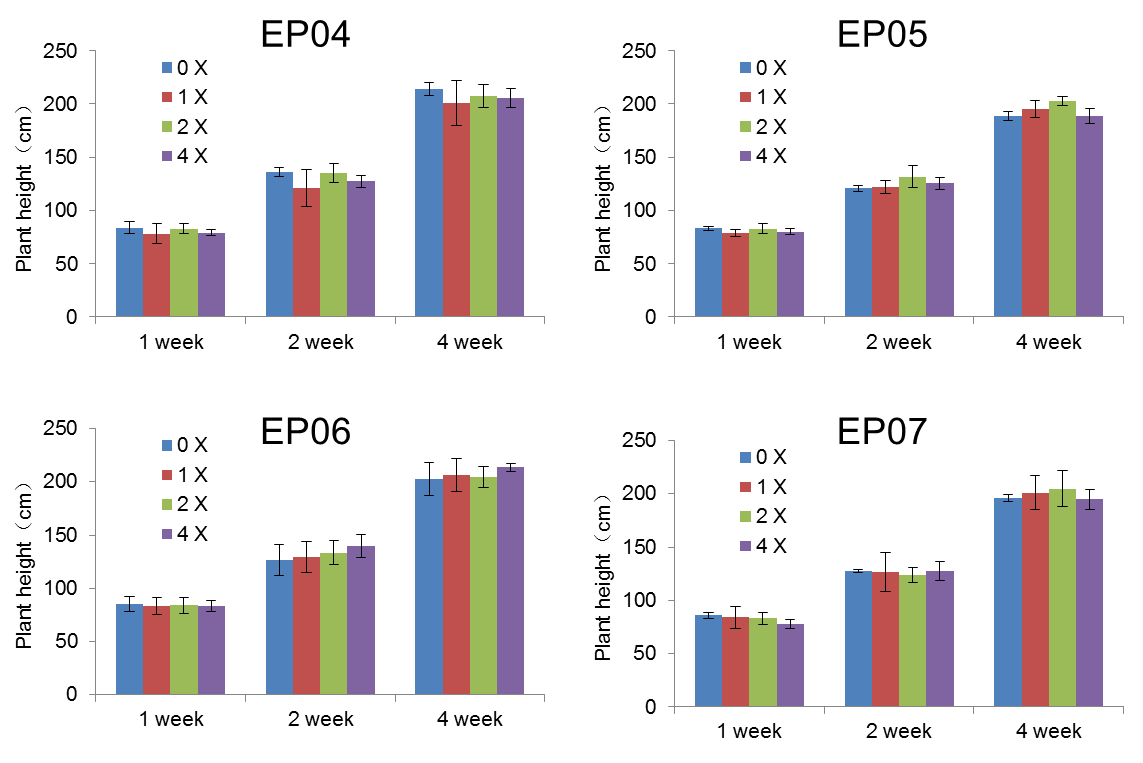
Supplemental Figure S6. Plant height of transgenic maize events at 1, 2, 4 week after spraying with 0, 1, 2 and 4 folds of the recommended field dosage of glyphosate.**
